# Autonomic Recovery Is Delayed in Chinese Compared with Caucasian following Treadmill Exercise

**DOI:** 10.1371/journal.pone.0147104

**Published:** 2016-01-19

**Authors:** Peng Sun, Huimin Yan, Sushant M. Ranadive, Abbi D. Lane, Rebecca M. Kappus, Kanokwan Bunsawat, Tracy Baynard, Min Hu, Shichang Li, Bo Fernhall

**Affiliations:** 1 Key Laboratory of Adolescent Health Assessment and Exercise Intervention, Ministry of Education, East China Normal University, Shanghai, China; 2 College of Applied Health Sciences, University of Illinois, Chicago, Illinois, United States of America; 3 Exercise and Cardiovascular Research Laboratory, Department of Kinesiology and Community Health, University of Illinois, Urbana-Champaign, Illinois, United States of America; 4 Guangzhou Institute of Physical Education, Guangzhou, China; University of Hull, UNITED KINGDOM

## Abstract

Caucasian populations have a higher prevalence of cardiovascular disease (CVD) when compared with their Chinese counterparts and CVD is associated with autonomic function. It is unknown whether autonomic function during exercise recovery differs between Caucasians and Chinese. The present study investigated autonomic recovery following an acute bout of treadmill exercise in healthy Caucasians and Chinese. Sixty-two participants (30 Caucasian and 32 Chinese, 50% male) performed an acute bout of treadmill exercise at 70% of heart rate reserve. Heart rate variability (HRV) and baroreflex sensitivity (BRS) were obtained during 5-min epochs at pre-exercise, 30-min, and 60-min post-exercise. HRV was assessed using frequency [natural logarithm of high (LnHF) and low frequency (LnLF) powers, normalized high (nHF) and low frequency (nLF) powers, and LF/HF ratio] and time domains [Root mean square of successive differences (RMSSD), natural logarithm of RMSSD (LnRMSSD) and R–R interval (RRI)]. Spontaneous BRS included both up-up and down-down sequences. At pre-exercise, no group differences were observed for any HR, HRV and BRS parameters. During exercise recovery, significant race-by-time interactions were observed for LnHF, nHF, nLF, LF/HF, LnRMSSD, RRI, HR, and BRS (up-up). The declines in LnHF, nHF, RMSSD, RRI and BRS (up-up) and the increases in LF/HF, nLF and HR were blunted in Chinese when compared to Caucasians from pre-exercise to 30-min to 60-min post-exercise. Chinese exhibited delayed autonomic recovery following an acute bout of treadmill exercise. This delayed autonomic recovery may result from greater sympathetic dominance and extended vagal withdrawal in Chinese.

***Trial Registration***: Chinese Clinical Trial Register ChiCTR-IPR-15006684

## Introduction

Altered cardiac autonomic function, an increase in sympathetic activity and a decrease in vagal activity, is associated with cardiovascular disease (CVD) morbidity and mortality [1]. Although autonomic function can be improved with chronic endurance training [2], acute exercise can induce an imbalance in the autonomic control of heart rate (HR) [3]. Importantly, alterations in autonomic function immediately after a single bout of exercise have been strongly associated with CVD morbidity [4].

Analysis of heart rate variability (HRV) [5] and baroreflex sensitivity (BRS) [6]have been verified as noninvasive and inexpensive methods for assessment of autonomic function. During exercise, there is a shift towards sympathetic dominance, resulting in HR acceleration caused by both increased sympathetic nerve activity and vagal withdrawal [7]. During exercise recovery, vagal reactivation is primarily responsible for HR decreases in the first min of recovery, followed by a withdrawal in sympathetic nerve activity after the first min of recovery [8]. The high frequency (HF) component of the HRV is mediated almost entirely by vagal modulation of the sinoatrial node directly associated with respiratory activity [9]. Although more controversial, the LF band is associated with baroreceptor-mediated regulation of blood pressure (BP) and reflects the mixed modulation of sympathetic and parasympathetic modulation [10]. The ratio of LF/HF reflects sympathovagal balance, and high values suggest a sympathetic predominance [9]. Activation of arterial baroreceptors via alterations in systemic arterial pressure results in reflexive changes in HR [11]. A decrease or increase in systemic arterial pressure initiates reflexive withdrawal or increase of cardiac vagal modulation, thus altering HR. Consequently, spontaneous BRS represents reflex cardiac vagal nervous modulation [12].

A previous study [13] reported that Caucasian populations have a higher prevalence of CVD when compared with their Chinese counterparts and CVD is associated with altered autonomic function. However, there is little information comparing autonomic function between Caucasian and Chinese population samples [14]. It is also unknown whether there is any difference in autonomic function during exercise recovery between Caucasians and Chinese. Given the higher CVD prevalence in Caucasians, it is possible that their autonomic function may be altered, but this is unknown. The present study investigated autonomic recovery following an acute bout of treadmill exercise between Caucasians and Chinese using HRV and BRS analyses at pre and 30-min and 60-min post- exercise.

## Material and Methods

### Ethics statement

The experimental procedure was approved by University of Illinois at Urbana-Champaign institutional review board and met the standards of the Declaration of Helsinki. Each participant was informed of the discomfort associated with acute exercise and provided written informed consent.

### Participants

**Fig 1:**
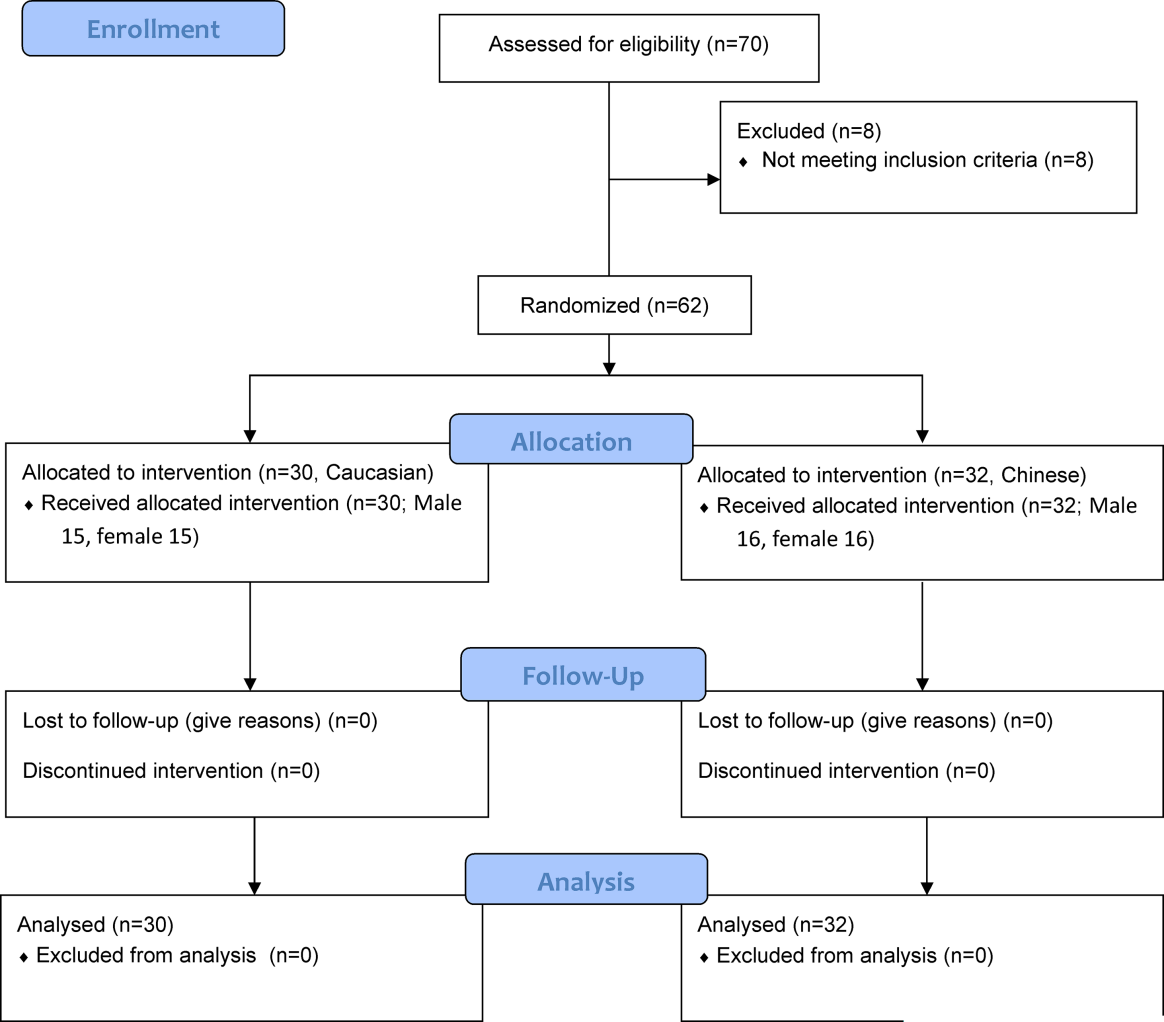
CONSORT Flow diagram.

Sixty-two (30 Caucasians, 32 Chinese, 50% male, respectively) moderately active participants between the age of 18 and 40 years volunteered for this study. (Fig 1) Activity level was assessed using the Lipid Research Clinics Questionnaire [15]. All participants were healthy, non-smokers, normotensive (systolic BP < 140 mmHg and diastolic BP < 90 mmHg) not obese (body mass index<30kg/m^2^), and free of any known cardiovascular or metabolic diseases as determined from a medical history questionnaire. Participants were self-defined as Caucasian or Chinese, if reporting that both parents were of Caucasian or Chinese descent. All participants, who residing in the cities of Urbana and Champaign, IL, were recruited via local advertisements. This research is conducted at the Exercise and Cardiovascular Research Lab of University of Illinois at Urbana-Champaign. The date range for participant recruitment was from February 24, 2011 to February 10, 2012.

### Study design

All participants reported to the laboratory on two occasions and the time interval between each visit was 48 hours-2weeks. Participants were instructed to abstain from caffeine, alcohol and vigorous exercise for 24-hours and were at least 3 hours post-prandial prior to testing. During the first visit, all participants completed a health history questionnaire and underwent a physical activity examination including measurements of height, weight and BP, peak aerobic capacity (VO_2_peak) and maximal heart rate (HRmax). During the second visit, all participants were required to rest in the supine position for a period of 10 min in a temperature-controlled room before testing. Then, HR, HRV, and BRS parameters were measured while participants were in the supine position for 5 min. Following pre-exercise data acquisition, participants underwent a supervised treadmill exercise bout. After the completion of the exercise protocol, participants resumed the supine position for recovery data acquisition. All measurements were obtained again 30-min [16] and 60-min [8] after exercise.

### Anthropometrics

Height and weight were measured as previously described [17]. Body mass index (BMI) was calculated as weight (kg) divided by height (m) squared. With the participants in a supine position, the intima-media thickness (IMT) of the right common carotid artery was measured by ultrasonography (SSD-α10, Aloka, Tokyo, Japan) using a 7.5-MHz linear-array probe and was defined as the distance between the leading edge of the lumen–intima interface to the leading edge of the media adventitia interface of the far wall. All measurements for the carotid IMT were made at end-diastole from an average of 5 measurements of a 10-mm segment obtained 1-2cm proximal to the carotid bifurcation using automated wall-tracking software.

### Peak aerobic capacity

Peak aerobic capacity was assessed using the Bruce treadmill protocol [18]. HR was measured using the Polar Heart Rate Monitor (Polar Electro, Woodbury, NY, USA). Expired air was analyzed by a Quark b2 breath-by-breath metabolic system (Cosmed, Rome, Italy). Rating of perceived exertion was assessed once every exercise stage and at peak exercise using the Borg scale [19]. The test was terminated when participants met two of the following four criteria: (1) a plateau in HR despite an increase in workload, (2) a plateau in oxygen uptake with an increase in work load, (3) the inability to maintain pace to keep up with the treadmill speed, and (4) a respiratory exchange ratio >1.1 [20]. Maximal heart rate (HRmax) was obtained to calculate the target HR during the exercise session.

### Acute exercise session

An acute bout of treadmill exercise session was performed at the laboratory. After 5 min of light warm-up on a treadmill, the participants performed 45 min of treadmill exercise at 70% of heart rate reserve. HRtarget = (HRmax—HRrest) × 70% + HRrest, where HRtarget and HRrest are HR during exercise session and at pre-exercise, respectively.

### HRV assessment

Beat-to-beat HR was recorded using three electrodes placed on the torso of the participant, in the supine position, continuously for 5 min. The ECG was collected online at a sampling rate of 1000 Hz by Analog to Digital Converter (MP100, BIOPAC Systems Inc., CA, USA) and stored offline on a computer for analysis at a later time. The recordings were checked visually to ensure that all QRS complexes on the ECG were correctly labeled. The HRV was analyzed in the frequency and time domains. In the frequency domain, the beat-to-beat fluctuations were transformed into frequency domains using Fast Fourier transformation, and the total power (TP), HF (0.15–0.4 Hz), LF (0.04–0.15 Hz) and LF/HF ratio were detected by an autoregressive model using WinCPRS software (Turku, Finland). In the time domain, the mean R–R interval (RRI) and root mean square of successive differences (RMSSD) were also calculated. The LF and HF power were transformed into normalized units as follows: nLF = LF power / TP-VLF power × 100 and nHF = HF power / TP-VLF power × 100 [21]. All data acquisition and post-acquisition analyses were carried out in accordance with standards put forth by the Task Force of the European Society of Cardiology and North American Society of Pacing and Electrophysiology [21]. Due to negatively skewed distributions, TP, HF, LF and RMSSD were normalized using natural log transformation prior to statistical analysis.

### BRS assessment

As reported in detail previously [22], with the participants in the supine position, beat-to-beat BP was monitored using a 5-min epoch via finger plethysmography (Finometer, FMS, Amsterdam, the Netherlands). The spontaneous cardiovagal baroreflex sensitivity was determined from the instantaneous and reflexive changes in inter-beat intervals (IBI) and systolic BP (SBP) [17]. Spontaneous breathing frequency (BF) was recorded according to the ups and downs of participants’ chest by manual counting. Any episode of three or more consecutive heartbeats in which the IBI and the corresponding SBP changed in the same direction (either up or down) was recorded. The slope of the regression line for each episode was calculated (WinCPRS), and the mean slope was an index of BRS [23]. Both up [BRS (up-up)] and down [BRS (dn-dn)] sequences were included in the final calculation of the spontaneous BRS. If the observed correlation coefficients were not statistically significant, results were discarded. Only sequences with correlations equal or greater than 0.80 were accepted.

### Statistical analysis

All data are presented as mean ± SE. A one-way Multivariate ANOVA was performed to determine racial differences in descriptive characteristics between Caucasian and Chinese groups. A 2 × 3 (2 race × 3 time-points) ANCOVA analyses with repeated measures were conducted on HR, HRV and BRS index variables to compare the differences between races and time. We also conducted a repeated measures analysis on the change values from pre-exercise (2 race × 2 time). When a significant race by time interaction was detected, *Bonferroni post hoc t-tests* were performed to determine where the difference occurred. Age was used as covariate. Significance was declared if p<0.05. Statistical Package for the Social Sciences (SPSS, Chicago, IL) version 17.0 was used.

## Results

Participant characteristics are presented in Table 1. Chinese participants were slightly older than the Caucasian counterparts (P<0.05). The body mass and height were significantly higher in Caucasians than Chinese (P<0.05), but no group difference was found in BMI. No group differences were found in IMT and VO2peak. No group differences were observed for HR at pre-exercise, maximum HR and target HR for exercise.

**Table 1:** Participants characteristics.

Variables	Caucasian (n = 30)	Chinese (n = 32)	P-value
Age, yr	24±4	28±4	< 0.001
Body mass, kg	68±9	62±10	0.013
Height, cm	172±10	165±8	0.004
Body mass index, kg/m^2^	23.1±2.5	22.5±2.6	0.295
VO_2_peak, ml/(kg·min)	47.0±9.6	43.3±6.3	0.086
Heart rate at rest, beats /min	64±13	68±11	0.174
Maximum heart rate, beats/min	193±10	195±8	0.360
Target heart rate, beats /min	154±10	157±8	0.116
Carotid artery intima-media thickness, mm	0.40±0.46	0.40±0.05	0.683

Values are means ±SE; n, No. of participants.

**Fig 2:**
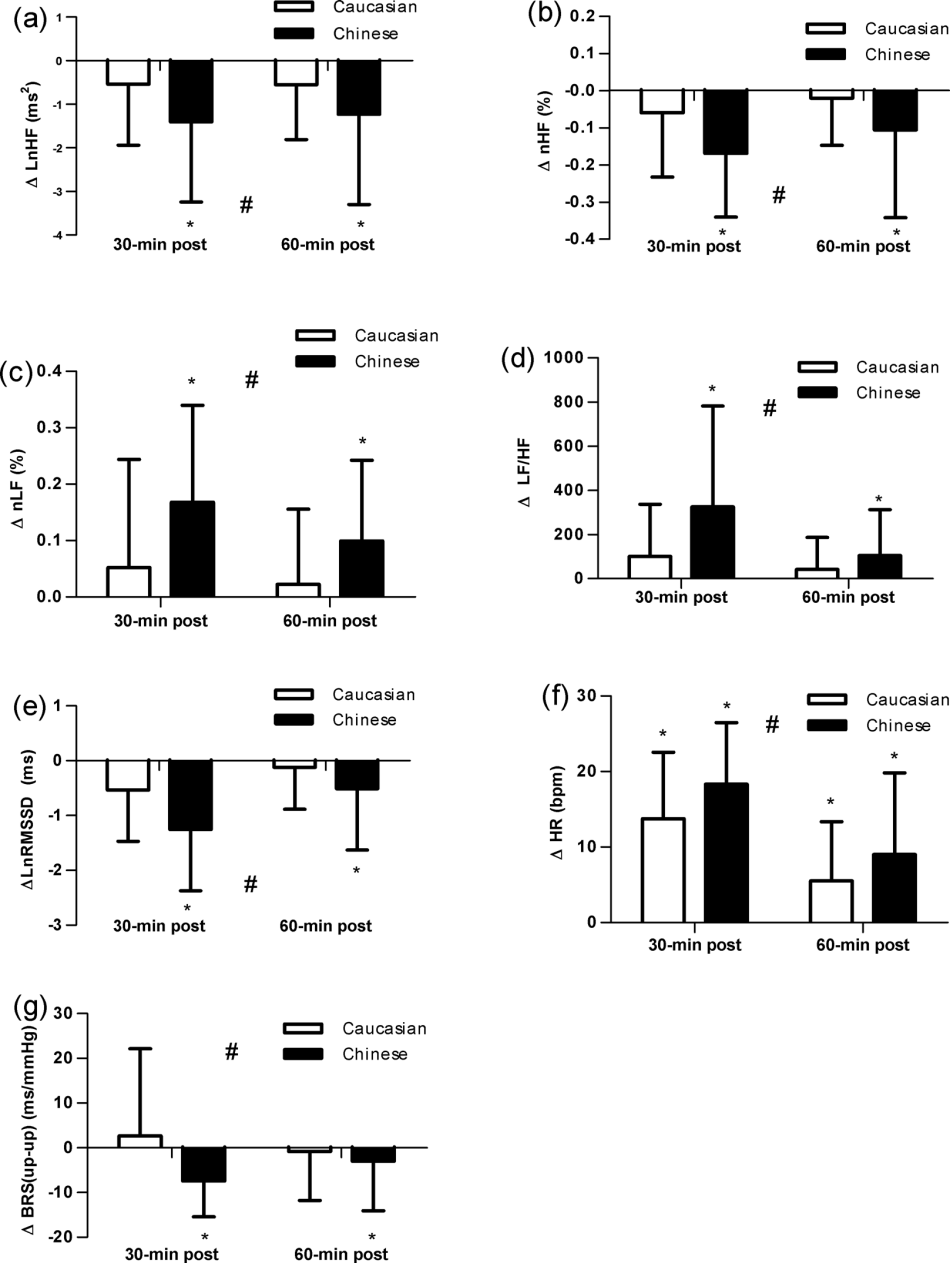
Change value from baseline for (a) LnHF, natural logarithm of high frequency (b) nHF, normalized high frequency (c) nLF, normalized low frequency (d) LF/HF, LF/ HF ratio (e) LnRMSSD, natural logarithm of RMSSD (f) HR, heart rate (g) BRS(up-up), Baroreflex sensitivity up-up, at 30-min and 60-min after exercise. Data are reported as mean ± SE (#significant race-by-time interaction; *significant change compared with baseline).

The changes in HRV parameters from the frequency domain before and after exercise are shown in Table 2. There was a significant race-by-time interaction for the changes in LnHF, nHF, nLF, and LF/HF from pre to 30-min and 60-min post-exercise (P<0.05). The change values of LnHF (ΔLnHF) and nHF (ΔnHF) were significantly greater in Chinese at 30-min and 60-min post-exercise (P<0.05). Although there was a trend up to 30-min and 60-min post-exercise to cause a decrease in ΔLnHF and ΔnHF in Caucasians, the reduction did not reached significance (Fig 2A and 2B). The change values of nLF (ΔnLF) and LF/HF ratio (ΔLF/HF) were significantly increased at 30-min and 60-min post-exercise in Chinese (P<0.05), compared with pre-exercise, but the significant increase was not shown in Caucasians during exercise recovery (Fig 2C and 2D). The changes in HRV parameters from the time domain before and after exercise are shown in Table 3. A significant race-by-time interaction was found in LnRMSSD, R-RI and HR (P<0.05). The change value of LnRMSSD (ΔLnRMSSD) was significantly greater in Chinese from pre to 30-min to 60-min post-exercise (P<0.05). However, the decrease was not significant in Caucasians at either 30-min or 60-min post-exercise (Fig 2E). There was an increase of change value of HR (ΔHR) (P<0.05), which was attenuated from 30-min to 60-min post-exercise, but the increase was greater in Chinese than in Caucasians both at 30-min and 60-min post-exercise. Furthermore, the HR at 60-min post-exercise were higher than pre-exercise in both Caucasians and Chinese (P<0.05) (Fig 2D). Table 3 demonstrates that the breathing frequency of Caucasian participants was similar to that of Chinese at pre-exercise and during recovery phase.

**Table 2:** Frequency domain measures of heart rate variability at baseline and recovery following exercise in Caucasian and Chinese.

Variables	Pre exercise	Post 30-min	Post 60-min	
LnTP, ms^2^				
Caucasian	8.07±0.91	7.34±1.23	7.95±0.95	Δ#
Chinese	7.95±1.31	6.47±1.27	7.39±1.16	
LnHF, ms^2^				
Caucasian	6.36±1.29	5.52±1.67	6.28±1.38	*Δ#
Chinese	6.31±1.78	3.98±1.97	5.36±1.73	
LnLF, ms^2^				
Caucasian	6.83±0.94	6.29±1.27	6.88±1.02	
Chinese	6.63±1.48	5.22±1.37	6.14±1.41	
nHF, %				
Caucasian	0.38±0.14	0.32±0.15	0.36±0.16	*Δ
Chinese	0.43±0.2	0.26±0.16	0.33±0.16	
nLF, %				
Caucasian	0.60±0.15	0.65±0.17	0.62±0.16	*Δ
Chinese	0.55±0.2	0.72±0.18	0.65±0.17	
LF/ HF				
Caucasian	192±115	292±226	233±168	*Δ
Chinese	192±194	517±528	296±257	

LnTP, natural logarithm of total power; LnHF, natural logarithm of high frequency; LnLF, natural logarithm of low frequency; nHF, normalized HF; nLF, normalized LF.

*P<0.05, significant difference from pre-exercise to 30-min and 60-min after exercise between Caucasians and Chinese.

Δ P<0.05, significant difference from pre-exercise to 30-min and 60-min.

# P<0.05, significant difference between Caucasians and Chinese.

**Table 3:** Time domain measures of heart rate variability at baseline and recovery following exercise in Caucasian and Chinese.

Variables	Pre exercise	Post 30-min	Post 60-min	
R-RI, ms				
Caucasian	963±209	820±175	873±170	*Δ#
Chinese	899±128	712±978	791±117	
HR, beats/min				
Caucasian	64±13	78±14	70±12	*Δ#
Chinese	68±11	87±10	77±13	
RMSSD, ms				
Caucasian	64.84±47.34	43.48±36.41	59.84±49.78	Δ
Chinese	69.47±90.9	17.87±15.42	40.3±38.5	
LnRMSSD, ms				
Caucasian	3.94±0.71	3.41±0.93	3.82±0.75	*Δ
Chinese	3.81±0.9	2.55±0.87	3.3±0.96	
BF, Hz				
Caucasian	0.26±0.09	0.3±0.09	0.28±0.08	Δ
Chinese	0.27±0.07	0.31±0.08	0.27±0.07	

R-RI, R-R interval; HR, heart rate; RMSSD, Square root of the mean of the sum of the squares of differences between adjacent NN intervals; LnRMSSD, natural logarithm of RMSSD; BF, breathing frequency.

*P<0.05, significant difference from pre-exercise to 30-min and 60-min after exercise between Caucasians and Chinese.

Δ P<0.05, significant difference from pre-exercise to 30-min and 60-min.

# P<0.05, significant difference between Caucasians and Chinese.

**Fig 3:**
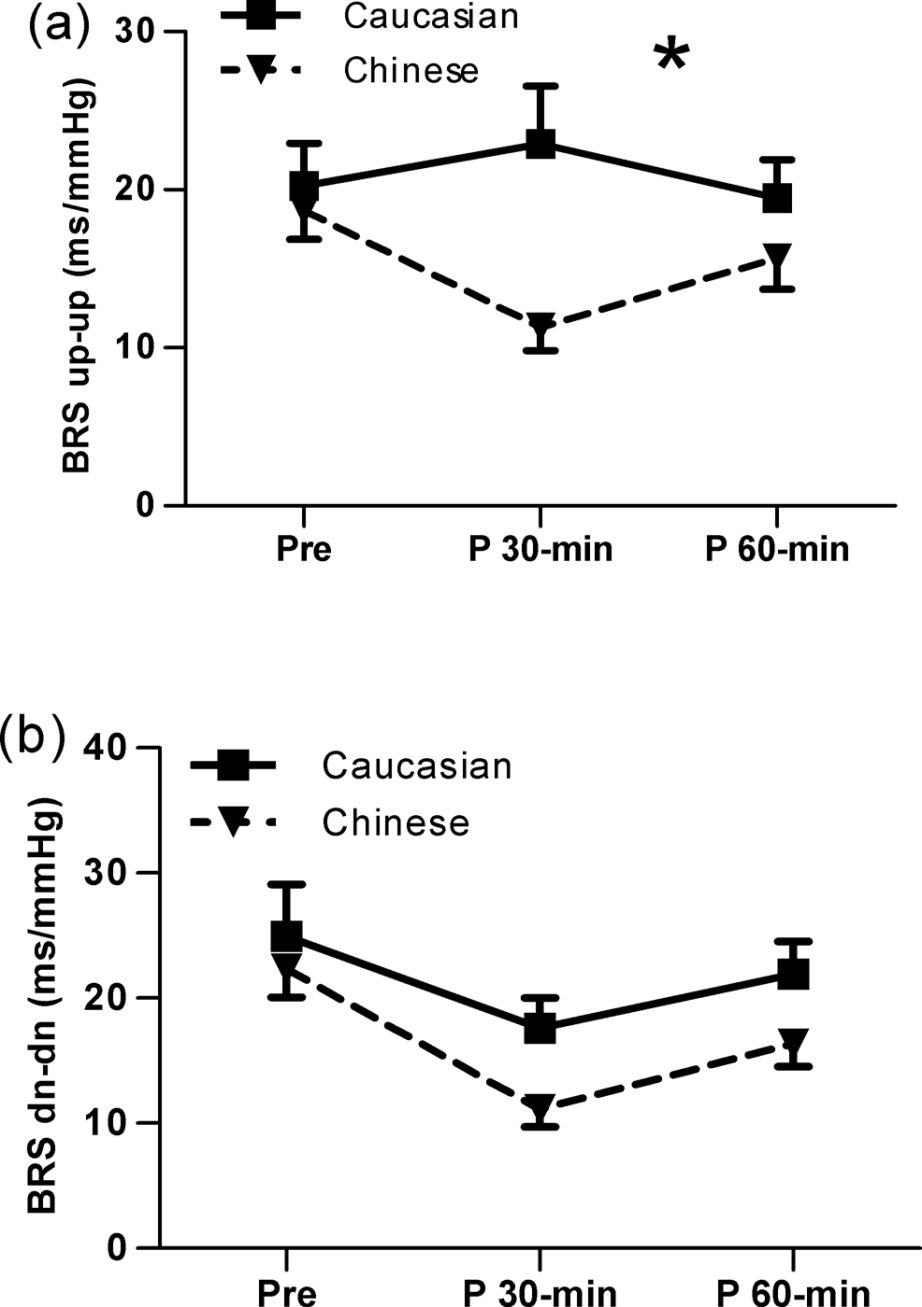
Baroreflex sensitivity (BRS) determined by the sequence method, pre and 30-min and 60-min post exercise in Caucasian and Chinese individuals. (a) BRS(up-up) showed a significant race by time interaction. *P<0.05 (b) There was no race-by-time interaction for the change in BRS(dn-dn).

The up-up and down-down sequences of BRS determined by the sequence methods are presented in Fig 3 There was no race-by-time interaction for the change in BRS (dn-dn) (Fig 3B), but a significant race-by-time interaction was found in BRS (up-up) (P<0.05, Fig 3A). The change in BRS (up-up) [ΔBRS(up-up)] was greater at 30-min and 60-min post-exercise in Chinese (P<0.05) than in Caucasians (Fig 2G).

## Discussion

This study examined autonomic function using, HRV and BRS in both young and health Caucasian and Chinese participants during exercise recovery from a single bout of treadmill exercise. The main finding of the present study is that LnHF, nHF and LnRMSSD, which reflected parasympathetic modulation, were significantly decreased, while nLF and LF/HF, which reflected sympathetic dominance, were significantly increased in Chinese compared with pre-exercise. The significant change was not found in Caucasians during exercise recovery compared with pre-exercise. Additionally, the decrease in the BRS (up-up) reflects a decreased reflex response to hypertensive stimuli during recovery in Chinese but not in Caucasians. Interestingly, although the incidence of CVD is higher in Caucasian compared to Chinese populations, and we speculated that autonomic function may be a contributor to this difference, our findings were contrary to our hypothesis. Our data suggest that the Chinese group exhibited “worse” autonomic function than the Caucasian group, and thus should be at higher risk for CVD. This concept will require further investigation in future studies.

### HRV regulation after exercise

Autonomic function is altered during exercise with an increase in sympathetic modulation and a decrease in parasympathetic modulation [3]. During exercise recovery, parasympathetic modulation is augmented, while sympathetic modulation gradually decreases towards pre-exercise [24]. Numerous human studies [25,26] show that neural activation of central command evokes a decrease in parasympathetic modulation and an increase in sympathetic modulation in response to acute exercise. Conversely, during exercise recovery, existing data have been inconsistent. Gladwell et al [3] reported an increase in parasympathetic modulation during the first 15 min of exercise recovery following three randomized 20-min cycling trials (moderate, hard or severe exercise intensities respectively). However, after the first 15 min, there was no difference in parasympathetic modulation from pre-exercise values. In contrast, Stuckey et al [24] found that a decrease in HF and an increase in LF/HF 60-min post-exercise following sprint interval exercise, indicating increased sympathetic dominance and decreased parasympathetic modulations. Such different recovery responses may be induced by the exercise stimulus different, suggesting interval exercise may induce longer autonomic recovery.

Our study showed no group differences in any pre-exercise spectral components of HRV, indicating similar resting autonomic function in Caucasians and Chinese. Previous studies [27,28] have shown that HRV is negatively correlated with age. Although there was a similar resting autonomic function in Caucasians and Chinese, Chinese participants were slightly older that Caucasian counterparts. Therefore, age was adjusted as covariate to avoid the possible influence of age on autonomic function during recovery stage after exercise. It is well known that HRV is negatively associated with IMT [29] and related to aerobic fitness level [30], also no group difference was found in IMT and VO2peak. The present findings showed that LnHF and nHF were depressed compared to pre-exercise values at both 30–min and 60-min post-exercise, suggesting parasympathetic modulation during exercise recovery was depressed for an hour in only Chinese, but not in Caucasians. Instead, parasympathetic modulation returned to pre-exercise values at 30-min post-exercise in Caucasians. Taken together, our findings indicate that parasympathetic modulation recovered much faster in Caucasians than in Chinese. The reduction of vagal modulation to the sinoatrial node was also indicated by the RMSSD of HRV [3], showing that Chinese showed attenuated parasympathetic recovery compared to Caucasians.

Given nLF represents relative contribution of the sympathetic modulation to the heart [31], the increase in nLF observed in Chinese suggested that an increase of sympathetic modulation during exercise recovery. This result supports the findings of Stuckey et al [24] showing that nLF was augmented until 60-min post-exercise. Interestingly, nLF was not altered in Caucasians during recovery, suggesting that Caucasians may exhibit faster sympathetic withdrawal than Chinese following recovery from exercise.

Despite controversies [32] and potentially lower reliability[33], the LF/HF ratio is generally indicative of sympathovagal balance. Previous studies [25,34] have shown that exercise produces an increase in LF/HF, indicating an increase in cardiac sympathetic dominance after exercise. Consistent with previous literature, our data shows an increase in LF/HF in Chinese during exercise recovery, suggesting an increase in sympathetic dominance. However, the LF/HF ratio at 60-min post-exercise did not return to pre-exercise in Chinese, indicating the recovery of sympathovagal balance would take more than 1 hour after acute treadmill exercise in Chinese. Conversely, at 30-min post-exercise, the LF/HF ratio was not significantly different from pre-exercise values in Caucasians, suggesting sympathovagal balance had been restored to pre-exercise in Caucasians at 30-min post-exercise. These findings suggest that Chinese exhibited delayed sympathetic withdrawal after acute treadmill exercise.

### HR regulation after exercise

It is interesting to note that HR recovery was not synchronized with the recovery of HF, LF, RMSSD and LF/HF in Caucasians; instead, HR did not completely return to pre-exercise at 60-min post-exercise. This suggests that, in addition to the autonomic nervous system, there may be other factors influencing HR recovery. White et al [35] suggested that increases in exercise workload-related HR are not caused by a total withdrawal of the parasympathetic nervous system followed by an increase in sympathetic tone. Circulating hormones [36] and catecholamines (NE and EPI) [37] may influence HR during exercise recovery. Along this line, Guyton et al [38] also reported that an elevation of catecholamines affects HR recovery after moderate to severe exercise. Perini et al [39] found a persistently elevated level of catecholamines after acute exercise. Unfortunately, these factors mentioned above were not measured in this study. The precise mechanism of the slower HR recovery than that in HRV index in Caucasians was unclear from the present study.

### BRS regulation after exercise

Alterations in BRS contribute to the reciprocal alterations of parasympathetic and sympathetic activity [12]. Previous studies show that BRS is decreased after acute exercise compared with pre-exercise values [17,26]. Stuckey et al [24] reported that BRS recovery was extended beyond 2 h following exercise. Our data also showed a decrease in BRS (up-up) in Chinese from pre to 30-min to 60-min post-exercise. However, the significant alteration in BRS (up-up) was not found in Caucasians at either 30-min or 60-min post-exercise, suggesting that sympathetic and parasympathetic modulations has completely returned to pre-exercise at 30-min post-exercise in this group. Thus, the restoration of autonomic function, inferred by BRS, was faster in Caucasians than in Chinese during exercise recovery.

There are several limitations to this study. We did not control for breathing rate during the assessment of HRV. Also, we measured the HRV and BRS parameters only at pre, 30-min and 60min post-exercise. The values of immediately after exercise or longer than 60 min during exercise recovery would have made our conclusion stronger. Our sample size was also relatively small and may not have been a representative sample of the population of either ethnicity. Finally, all of the participants of Chinese decent lived in the US, thus may have adopted different lifestyles that individuals living in China, which may have influenced our results.

In conclusion, we investigated cardiac autonomic function in response to acute treadmill exercise and observed a shift towards greater sympathetic dominance in Chinese than in Caucasians during exercise recovery, suggesting that autonomic recovery is delayed in Chinese following acute treadmill exercise.
